# Editorial: Microbial Role in the Carbon Cycle in Tropical Inland Aquatic Ecosystems

**DOI:** 10.3389/fmicb.2017.00020

**Published:** 2017-01-19

**Authors:** André M. Amado, Fábio Roland

**Affiliations:** ^1^Limnology Laboratory, Department of Oceanography and Limnology, Universidade Federal do Rio Grande do NorteRio Grande do Norte, Brazil; ^2^Aquatic Ecology Laboratory, Department of Biology, Universidade Federal de Juiz de ForaMinas Gerais, Brazil

**Keywords:** metabolism, lakes, lagoons, rivers, latitudinal gradients, microorganisms, plankton, microbial ecology

Microorganisms have been recognized as central to nutrient mineralization and recycling in aquatic ecosystems since Lindeman's groundbreaking work on the trophic-dynamic aspect of ecology (Lindeman, 1942). Since the seventies, the development of new analytical technologies led to important conceptual perspectives, such as the microbial loop and the microbial food web (as summarized elsewhere, e.g., Cotner and Biddanda, 2002; Weisse, 2004), which have been important to understanding connections between microbially-mediated allochthonous and autochthonous organic matter decomposition and carbon dioxide (CO_2_) concentrations and fluxes to the atmosphere (e.g., Cole et al., 2007; Berggren et al., 2012; Fonte et al., 2013). Currently, one of the main foci of microbial ecologists is to open the “microbial playbill” so that we can better understand who is doing “what” and “when” in ecosystem “plays” (Logue et al., 2015).

It is well-documented how seasonal variation of temperature, light incidence, and precipitation affects microbial metabolism (e.g., Simon and Rosenstock, 1992; Berggren et al., 2010) in high latitude ecosystems. Considering that temperature and light incidence are less variable and remain high year-round in the tropics (Lewis, 1996), one could expect differences in metabolic processes among the latitudinal regions (Farjalla et al., 2009) and consequent effects on microbial respiration and carbon emissions (essentially CO_2_ and methane) to the atmosphere. On the one hand, more intense metabolic processes are expected in lower latitudes. On the other hand, regional (e.g., flood pulse) or local (e.g., landscape characteristics) environmental conditions could be more relevant regulators of microbial metabolism than global factors (e.g., temperature, etc.). For instance, small rather than large planktonic organisms predominate at the base of microbial food webs of tropical aquatic ecosystems (i.e., pico- vs. nano-plankton) in comparison to temperate lakes, which typically means a higher flow of carbon through the microbes in the tropics than in temperate regions (Roland et al., 2010; Sarmento, 2012). Yet, certainly several new microbial ecology fundamentals will arise from asking questions that remain poorly understood, such as: (1) Does the current knowledge derived mostly from temperate ecosystems hold for tropical ecosystems? and (2) Can tropical ecosystems be good models to predict the changes in microbial metabolism and carbon cycling in temperate aquatic systems in light of climate warming scenarios?

The aim of this research topic—*Microbial role in the carbon cycle in tropical inland aquatic ecosystems*—was to provide a selection of studies that look at the wide variety of aspects of tropical microbial ecology including barely covered outlines (such as viruses–bacteria interactions or hydrodynamic events driven microbial communities) and their role to the carbon cycle. This research topic has documented 13 contributions that advance our knowledge on the microbial responses to natural latitudinal gradients, spatial and temporal patterns within and between ecosystems, and temperature and nutrient effects on microbial processes in the water and in the sediment. In order to better compile the information, the contributions were grouped in the following paragraphs as: (a) how physical and chemical variables (e.g., temperature and nutrients) affect carbon metabolism via microbes; (b) how latitudinal variation affects metabolism in the microbial food web components, and (c) how changes in environmental forces (such as flood pulse) affect microbial interactions.

Two meta-analysis studies focused on bacterial metabolism and top-down control and how they vary across latitudinal gradients. One study demonstrated that bacterial biomass production (BP) and respiration (BR) are higher while bacterial growth efficiency (BGE) is lower in the tropics (Amado et al.). Additionally, the other study demonstrated that bacterial and heterotrophic flagellate (HNF) abundance were lower in tropical than in temperate ecosystems (Segovia et al.). Furthermore, the authors still showed that the coupling between predator and prey did not differ between tropical and temperate zones, suggesting that other factors, such as the higher temperature or even HNF top-down control could be responsible for higher bacterial loss rates in the tropics.

One study that manipulated temperature and nutrients in tropical humic coastal lagoons (Scofield et al.) recorded increased BR and decreased BP and BGE with increasing temperature, confirming the results reported by Amado et al., and showing that temperature can be a valuable carbon metabolism predictor on a regional scale. Similarly, nutrient manipulations resulted in different metabolic responses and CO_2_ saturation patterns among lagoons, indicating that temperature effects are modified by local ecosystem conditions (Peixoto et al.). Additionally, another study performed in an eutrophic reservoir (Almeida et al.), observed net heterotrophy, net CO_2_ emission, and high ebullition and diffusive methane (CH_4_) emissions. Despite high primary production rates, the shallow nature of the ecosystem, the high temperatures, and accumulated organic matter contributed to high decomposition rates. These three studies supported the idea that local factors are strong drivers of aquatic carbon metabolism at low latitudes.

The Amazon River basin is inserted in a low-latitude zone and it is recognized as a hotspot for freshwater CO_2_ emissions globally (Raymond et al., 2013; Abril et al., 2014). The flood pulse is the major ecological force affecting carbon-related processes and organisms in this region (Amado et al., 2006; Melack and Coe, 2012). Indeed, the flood pulse was the main driver for bacterial metabolism and carbon mobilization in rivers due to changes in the organic matter origin and quality (Vidal et al.). Moreover, one study in floodplain lakes of a large Amazon tributary contained bacterial and viral abundances that increased with increasing distance from the Amazon River (Almeida et al.). The longitudinal gradient was attributed to a backwater effect caused by the Amazon River that increased water turbidity and decreased organic carbon quality (availability) and concentration in the floodplain lakes downstream, which might also reduce the carbon flow through to the microbial food web.

Three studies evidenced that there is a great deal of heterogeneity among coastal lakes in the subtropical region. One study using seasonal and diel approaches in a coastal lagoon in southern Brazil (Peri Lagoon, Santa Catarina state), showed that precipitation and temperature, as well as bacterial abundance were the main regulators of CO_2_ concentration in the water (Schmitz Fontes et al.). On the other hand, the bacterial community and activity in another coastal subtropical lagoon (Laguna de Rocha, Uruguay), was carbon-limited and was mainly driven by organic matter inputs from the watershed or dilutions by the seawater (Alonso et al.). This resulted in shifts in the microbial community and the microbial food web through HNF and virus increase. In addition, the dominance of wetlands and aquatic macrophytes reduced microbial carbon metabolism and CO_2_ concentration in the water when compared to the pelagic areas (i.e., higher depths and no macrophytes presence) creating heterogeneous conditions to carbon processing within the lake in a subtropical coastal lagoon in southern Brazil (Lake Mangueira, Rio Grande do Sul state; They et al.). Subtropical lakes are somewhat of a transition type of ecosystem, which consistently share regulation of microbial and carbon processes by local/regional environmental factors with tropical ecosystems, and by climate-driven seasonal environmental factors with temperate ecosystems.

Sediment metabolism has been extensively demonstrated to be relevant to the carbon balance in deep temperate lakes (e.g., Jonsson et al., 2001), but was poorly studied in tropical inland waters. Cardoso et al. observed high mineralization rates in the sediment of a mesotrophic tropical hydroelectric reservoir, which has driven the CO_2_ variation in the water column and high internal spatial variation in the respiration. Also, Canterle et al. observed both spatial and temporal variation in the microbial CO_2_ and CH_4_ production and emission in the sediment of a subtropical shallow wetland ecosystem. Additionally, Liengaard et al. detected high emission of nitrous oxide (N_2_O) in the largest wetland in the world (Pantanal, a pristine tropical wetland) providing up to 1.7% of global N_2_O emissions, as result of high organic matter availability processed via anaerobic metabolism. These findings are especially relevant taking the climate warming scenario. The intensification of biological processes may increase greenhouse-gas emissions 2–4 times more in the tropics than in temperate sediments (Marotta et al., 2014).

The articles presented in this research topic investigate several aspects of microbial interaction with carbon cycling in tropical inland waters and produced some evidence that it can change across the latitudinal gradient as follows: (a) higher temperature in the tropics enhances bacterial carbon processing and reduces BGE; (b) the energy flow through the microbial food web in the tropics may be either reduced due to low BGE, or enhanced due to a possible high top-down control of HNF; and (c) as in temperate ecosystems it seems consistent that nutrient availability is also a key component of the aquatic metabolism and carbon processing in tropical ecosystems, but local or regional regulation may frequently be equally or even more relevant than globally controlled environmental factors. Nonetheless, several gaps still remain. For example, is absolutely pertinent a better understanding regarding the controls of trophic cascade relationships (e.g., top-down control of bacteria by HNF) in the microbial food web and how landscape conditions (e.g., land use) affect carbon processing in aquatic ecosystems. Certainly, many opportunities exist for research focusing on the relationship of carbon and the microbial components of freshwater. As we commented before, we expect that these papers will stimulate new discussions and investigations to cover the great diversity of ecological processes that still need to be addressed and help formulate more robust general patterns for tropical freshwaters.

## Author contributions

All authors listed, have made substantial, direct and intellectual contribution to the work, and approved it for publication.

## Funding

This research topic received support from National Council of Technological and Scientific Development (CNPq—Brazil) specifically to AA under the grant # 475537/2012-2 and to FR under the grant # 309180/2013-9.

### Conflict of interest statement

The authors declare that the research was conducted in the absence of any commercial or financial relationships that could be construed as a potential conflict of interest.
